# quantGenius: implementation of a decision support system for qPCR-based gene quantification

**DOI:** 10.1186/s12859-017-1688-7

**Published:** 2017-05-25

**Authors:** Špela Baebler, Miha Svalina, Marko Petek, Katja Stare, Ana Rotter, Maruša Pompe-Novak, Kristina Gruden

**Affiliations:** Department of Biotechnology and Systems Biology, National Institute of Biology, Ljubljana, 1000 SI Slovenia

**Keywords:** Quantitative molecular biology, Quantitative PCR, Nucleic acid quantification, Web application, Decision support system

## Abstract

**Background:**

Quantitative molecular biology remains a challenge for researchers due to inconsistent approaches for control of errors in the final results. Due to several factors that can influence the final result, quantitative analysis and interpretation of qPCR data are still not trivial. Together with the development of high-throughput qPCR platforms, there is a need for a tool allowing for robust, reliable and fast nucleic acid quantification.

**Results:**

We have developed “quantGenius” (http://quantgenius.nib.si), an open-access web application for a reliable qPCR-based quantification of nucleic acids. The quantGenius workflow interactively guides the user through data import, quality control (QC) and calculation steps. The input is machine- and chemistry–independent. Quantification is performed using the standard curve approach, with normalization to one or several reference genes. The special feature of the application is the implementation of user-guided QC-based decision support system, based on qPCR standards, that takes into account pipetting errors, assay amplification efficiencies, limits of detection and quantification of the assays as well as the control of PCR inhibition in individual samples. The intermediate calculations and final results are exportable in a data matrix suitable for further statistical analysis or visualization. We additionally compare the most important features of quantGenius with similar advanced software tools and illustrate the importance of proper QC system in the analysis of qPCR data in two use cases.

**Conclusions:**

To our knowledge, quantGenius is the only qPCR data analysis tool that integrates QC-based decision support and will help scientists to obtain reliable results which are the basis for biologically meaningful data interpretation.

**Electronic supplementary material:**

The online version of this article (doi:10.1186/s12859-017-1688-7) contains supplementary material, which is available to authorized users.

## Background

The immense potential of quantitative molecular biology in life sciences is challenged by inconsistent approaches for control of errors in the final results. Due to its performance characteristics and general applicability, quantitative PCR (qPCR) has become the golden standard method for the quantification of nucleic acids. Although with the help of laboratory automation, qPCR data generation has become easy and fast, quantitative data analysis and interpretation is still not trivial due to several factors that can influence the final result. To ensure high quality of results and allow for potential reproduction of experiment, the Minimum Information for Publication of Quantitative Real-Time PCR Experiments (MIQE) guidelines have been proposed [1].

qPCR is used to measure the quantity of target DNAs in a given sample through repeated cycles of DNA amplification. The cycle at which the observed amplification-derived fluorescence first exceeds a certain threshold is called the quantification cycle (Cq). The analysis starts with the examination of the amplification curves and initial assessment of their quality, followed by the determination of the C_q_ values, which are further used for the quantification of the nucleic acids. It can be performed by either a standard curve or a comparative approach (formerly referred to as “absolute” and “relative” quantification, respectively). Both approaches are relative, but each is based on its own assumptions [1]. In the standard curve approach, the number of target DNA molecules in the sample is calculated using a calibration curve of serially diluted DNA standards of known concentrations. The calibration curve presents a linear relationship between the C_q_ and the logarithm of the initial amount of template DNA. Test sample copy numbers are calculated from the linear regression of the standard curve, assuming equal amplification efficiencies for the standard and test samples [2]. When reference materials with known contents are available, the outcomes are absolute copy numbers [3] whereas when the copy numbers of the targets in the standards are not known, relative standard curves can be used to determine copy numbers ratios between different samples [4]. The second approach, comparative quantification, is based on determining the fold-differences in the expression of the target in relation to the reference gene. The most popular, comparative threshold cycle method (ΔΔC_q_) relies on a direct comparison of the C_q_ values and assumes equal and 100% efficiencies of the target and the reference gene. However, the amplification efficiencies between different genes analysed can differ which makes the ΔΔC_q_ method unsuitable in many cases [5]. Consequently, modifications that allow for amplification efficiency correction have been developed [1]. Although they do not perform as accurate as the standard curve approach [3], they can be applicable in research applications where high accuracy is not needed.

The efficiency of PCR amplification is considered as one of the most important parameters in qPCR analysis, as it strongly influences the final result [5, 6]. The efficiency is defined as the fraction of target molecules that are copied in one PCR cycle. Deviations from an optimal 100% efficiency are observed as inhibition, caused by the presence of inhibitory components, or over-amplification, caused by compound or structural conformation changes during the PCR [6]. Most common and broadly accepted way of efficiency determination is from the slope of a standard curve using linear least squares regression [1] where the preciseness of the efficiency estimate is affected by qPCR platform, the number of replicate reactions and serial dilution volume [7]. Recently, robust regression methods were shown to present a reliable alternative because they are less affected by outliers [8]. Alternatively, the efficiency can be calculated from the fluorescent increment in single amplification curves which were shown to be less accurate (reviewed in [9]) and they also require an additional step in the analysis that is sometimes cumbersome and impractical. The efficiency of the amplification is highly dependent on primer sequence and therefore the assumption of most quantification algorithms is that PCR efficiency is assay-dependent and sample-independent [10]. Yet, it is not uncommon that individual samples originating from different or even same matrix have different amplification efficiencies [6] which can result in quantification inaccuracies [11]. A simple control of efficiency in individual samples can be performed by analysing two dilutions of the same sample [6].

Normalization controls for variations in extraction yield, reverse-transcription yield and efficiencies of amplification, thus enabling comparisons of nucleic acid concentrations across different samples. Various normalization strategies and reference genes selection algorithms have been proposed with the common guideline that several validated reference genes should be used for normalization (reviewed in [12]).

Although numerous commercial and open-access software tools for the analysis of qPCR data exist (see [13] for a recent review), they lack quality control (QC) of the final result that would aid the researcher in interpreting it. We have developed the web application quantGenius (http://quantgenius.nib.si), the only qPCR data analysis solution that integrates a QC-based decision support system (DSS). Among other features, it includes a control of inhibition in individual samples which is extremely useful when working with difficult samples, such as environmental or plant samples. In this way, it helps the scientist to obtain reliable results in a fast and high-throughput manner and thus provides the basis for biologically meaningful data interpretation.

## Implementation

Front-end of the web application is built in HTML, CSS and JavaScript. Back end is written in PHP with extensive use of Laravel framework. The data is stored and managed using MySQL relational database management system (Additional file 1). The application is fully functional in most popular web browsers (Chrome, Internet Explorer 9+ and Firefox) with enabled JavaScript.

The most recent quantGenius release is available at http://quantGenius.nib.si. The source code for quantGenius is freely available under the GNU General Public License version 3.0. All the application functionalities are freely available without login or registration. Nevertheless, registration and login option have been implemented for users that wish to keep their datasets for later analysis.

In the application, data are organised as experiments, containing data for all the assays that were analysed in a sample set (see screenshot in the Additional file 1).

**Fig. 1:**
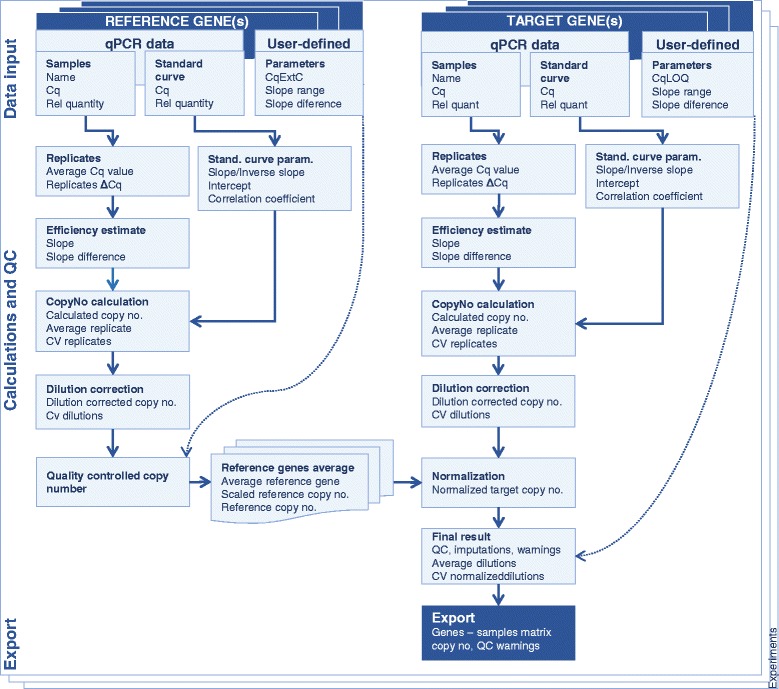
quantGenius workflow. The data for the target and reference genes is imported and quality controlled. Relative copy numbers are calculated using standard curve parameters and normalized. The final results are exported as a gene-sample matrix. The calculation steps are marked with bold, while the calculated parameters are listed below with regular letters. All the formulas used for calculations (Equations 1–25) are available in the Additional file 2. Abbreviations: Cq – quantification cycle, Rel. - relative no.- number, Std. – standard, QC – quality control, CV - coefficient of variation, CqExtC – Cq value of the extraction control, CqLOQ – Cq value of the limit of quantification

The quantGenius workflow features three main steps: 1) data import, 2) interactive calculation of target and reference genes copy numbers and normalization to reference genes with implemented QC-DSS and 3) export of final results in a gene-sample matrix format (Fig. 1). quantGenius enables a transparent overview of all calculations, including intermediate values and mathematical formulas used as well as QC-based decisions. All the formulas used for calculations are available in the Additional file 2 (Equations 1–25) and a detailed user manual is available on the application website.

## Results

### Platform-independent and consistency-checked data input

The application’s input is qPCR machine- and chemistry- independent. For each tested assay, sample names, C_q_ values and relative copy numbers (based on sample dilutions) are imported by pasting pre-formatted tab-delimited data into the input form. In this way, sample data analysed with one or two sample dilutions and any number of technical replicates can be processed. Data for the standard curve, which can be either actual or relative copy numbers, are imported separately (Additional file 3).

Standard output files of the microfluidic qPCR platform BioMark (Fluidigm) can be converted to a format suitable for the import using the “Fluidigm data prep tool”, available on the quantGenius website.

All imported data are automatically checked for consistency (i.e., that the sample names, replicates and a number of dilutions are consistent between the target and reference genes) to prevent wrong calculations due to incorrect imports (for example copy-pasting errors).

### Copy number calculation and normalization to reference genes

In quantGenius, a standard curve quantification approach is implemented, which allows for the calculation of comparable copy numbers on multi-plate experiments, when the same standard curve is used on all plates. For optimal transparency of the process, the calculations are performed in several steps (Fig. 1, Additional file 2), differing slightly whether simple (one-dilution) or two-dilution analysis is selected. Based on the standard curve parameters (Additional file 2, Equations 3-8), sample target and reference gene copy numbers are calculated (Additional file 2, Equation 11). In the next steps, replicate copy numbers are averaged and sample dilution is taken into account (Additional file 2, Equations 13, 14).

**Fig. 2:**
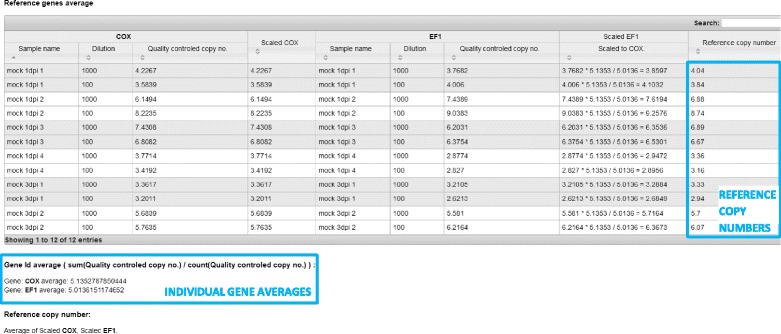
Screenshot of the calculation of the reference copy numbers. An example of two reference genes (*COX* and *EF*-*1*) is shown. The second imported reference gene (*EF*-*1*) is scaled to the average of the first reference gene imported and the average of both values is calculated. The calculations are performed for each dilution separately

Target gene copy numbers are normalized to reference gene copy numbers, or in the case of several reference genes, to their average (Fig. 2; Additional file 2, Equations 17–20). To avoid unequal contribution of the individual reference genes and to allow for quantification in the cases where data for one of the reference genes is missing due to QC issues, all the reference gene copy numbers are scaled to the average of the reference gene that was imported first (Additional file 2, Equation 18).

### User-guided quality control-based decision support system

**Fig. 3:**
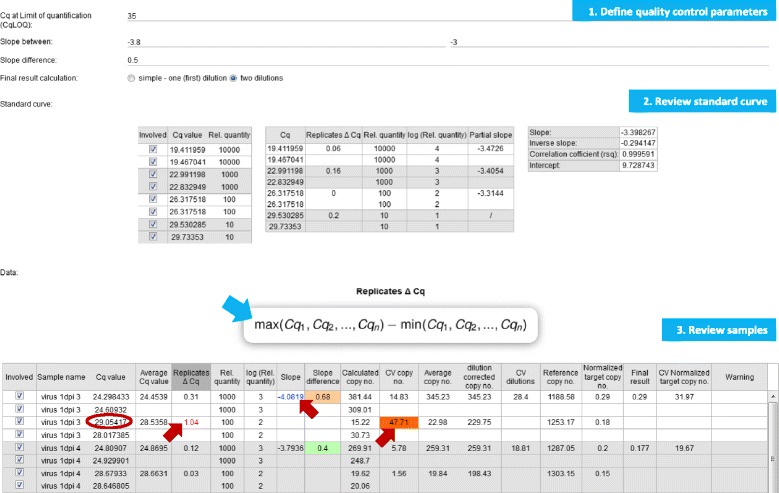
Screenshot of the individual gene calculations. The calculations are done in three steps. 1) QC parameters CqLOQ, CqExtC, slope range, and slope difference and calculation mode are defined by the user. 2) Standard curve is reviewed for possible outlier reactions. 3) Sample reactions are reviewed. The pipetting error (*red circle*) causes deviations from the predefined QC parameters (*red arrows*). All formulas used for the calculations can be viewed (*blue arrow*)

The unique and novel feature of the presented application, quantGenius, is the implementation of an easy-to-use QC-based DSS that enables robust analyses of quantitative biology data. It includes all critical parameters of qPCR QC, such as technical pipetting errors, nucleic acid extraction and reverse transcription yields, estimations of the detection and quantification ranges of the assays as well identification presence of inhibitors in the individual samples [3]. Several QC parameters are calculated at different steps of the workflow (Fig. 1, Additional file 2, Equations 2, 4, 5, 8–10, 13, 15, 22). The QC stringency is user-controlled, based on the level of accuracy required for particular application (Fig. 3). By changing the QC parameters all the data are instantly recalculated. Moreover, the “clone experiment” option allows for analysis of the same experiment with different QC parameters and thus direct comparison of the effects that the parameter settings changes have on the final results.

quantGenius enables a transparent overview of all QC-related issues and decisions. Highlighting of the values that fall out of the pre-defined QC thresholds enables the identification of the pipetting errors in the standard curve or target sample reactions as well as standard curve dilutions that are out of the quantification range which should all be manually removed by the user (Fig. 3).

**Fig. 4:**
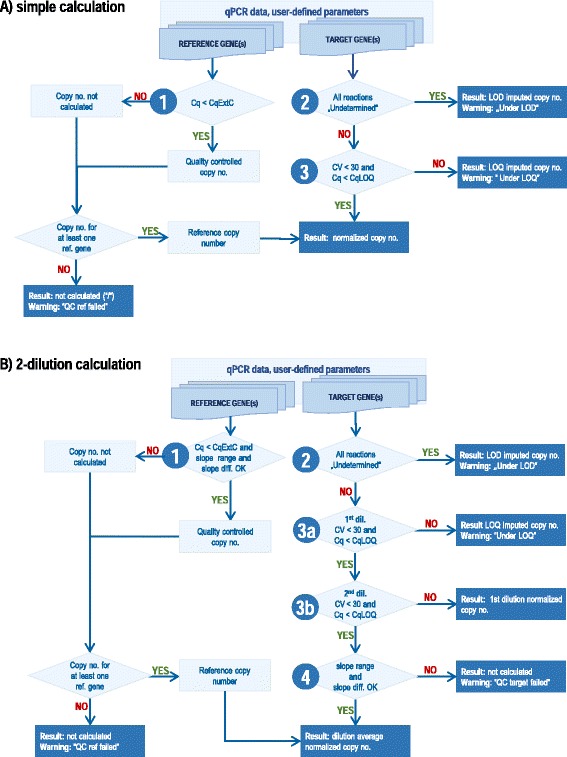
quantGenius quality control-based decision support system (DSS). Decision tree case of (**a**) simple (one-dilution) calculation and (**b**) two-dilution calculation. The following QC control steps are implemented hierarchically: 1) extraction control, 2) limit of detection 3) limit of quantification, and 4) individual sample efficiency of amplification control. Based on the DSS, the final result is calculated (*blue boxes*), modified (*orange boxes*) or not given (*red boxes*) and warnings are issued. Abbreviations: Cq – quantification cycle, CqExtC – Cq value of the extraction control, no. – number, CqLOQ – Cq value of the limit of quantification, dil. – dilution, QC – quality control, CV - coefficient of variation

Based on the implemented DSS, the final result will be, in the case that the data is out of the quantification range, imputed or, if the calculated numbers are considered unreliable, not given. In both cases, warnings are issued, notifying the user about the QC issues. The decision tree slightly differs depending on whether simple (one-dilution) or two-dilution calculation approach is chosen (Fig. 4) and hierarchically takes into account the following factors:

*Extraction control*. For each reference gene assay, C_qExtC_, a C_q_ value indicating a valid nucleic acid (DNA or RNA) extraction procedure is defined by the user ensuring that only good quality data is used for calculations. By default, the C_qExtC_ is set to 34, therefore rarely affecting the quantification. Based on the assumption that the reference genes are highly expressed, the users can, however, lower this threshold to identify outlier samples. If all the reference genes fail this criterion, the target gene final result is not calculated (Fig. 4).

*Limit of quantification (LOQ)*. For each target gene assay, the C_q_ at the LOQ (C_qLOQ_), specifying the lower limit of the quantification of the assay is defined by the user, either based on previous in-house validation data (if available) or estimated from the experiment’s standard curve. On the other hand, the LOQ can be recognized by quantGenius as high variability (CV > 30) between the replicates’ copy numbers, arising from pipetting stochasticity, assuming that the true pipetting errors have previously been manually removed. In the simple calculation, the final result for samples below LOQ is imputed based on the C_qLOQ_ and all sample reference gene data (Fig. 4a, Additional file 2, Equation 24). In the two-dilution calculation, the LOQ QC step is performed in two steps: a) if the first dilution (less diluted reactions) is under LOQ, the final result is calculated as in the simple calculation (described above), b) if only the second dilution (more diluted reactions) is under LOQ, the first dilution is used for the calculation of the final result (Fig. 4b).

*Limit of detection (LOD)*. If all reactions of the sample for a target gene are missing C_q_ values, indicating that the target DNA levels in the sample are under the LOD of the assay, then the final result is imputed based on the C_qLOQ_ copy numbers of all sample reference gene data (Fig. 4, Additional file 2, Equation 25). The final result is, therefore, a very small number (lower than the LOQ-imputed value but not zero) which makes further data analyses possible without additional data imputation. LOD imputation is performed only for target genes, as the reference genes must be present well above the LOQ.

*Individual sample amplification efficiency control*. This QC step is implemented only in the case of two-dilution calculation and is used to identify outlier samples with apparently inappropriate amplification efficiencies as compared to the one of the standard curve [6]. If the individual sample slope (Additional file 2, Equation 9) falls out of the pre-defined slope range or its difference from the standard curve’s slope (Additional file 2, Equation 10) is bigger than the pre-defined maximum slope difference, the reference or target gene copy numbers are not calculated for this sample (Fig. 4b).

### Export enabling further data analysis

All the data, imported sample names, quantities and C_q_ values, intermediate calculations and QC parameters as well final results are available for the export from quantGenius to allow for further analysis and visualization in third-party software tools. All the data per individual gene can be exported in Excel (.xls) format (see example in Additional file 4). On the other hand, final results for all the target genes in the experiment can be also exported in a form of a sample-gene matrix in tab-delimited.txt or.xls formats. In the latter, the results are complemented with the QC warnings, so the user can distinguish between values, calculated directly from the sample data or the imputed values.

### Comparison of features with advance qPCR analysis software tools

The quantification approach and crucial QC features of quantGenius were compared with similarly advanced software tools for qPCR data analysis: REST [14], one of the first software tools for qPCR analysis, two popular commercial packages qBASE+ (Biogazelle NV, [10]) and GenEx (MultiD Analyses AB) as well as an open source tool DAG Expression [15], one of the rare tools that uses standard curve based quantification (Table 1). It is important to note that the compared software tools have additional features that are not included in quantGenius, such as qualitative QC parameters (positive and negative controls, control of genomic DNA removal etc.) or further steps in the data analysis pipeline such as statistical analysis, graph plotting etc. These features were not included in quantGenius as it is focused on the quantification aspect of the qPCR data analysis pipeline.

**Table 1:** Comparison of selected features of quantGenius and other software tools

Analysis tool/Feature	Quantification method	Multiple reference genes	Quality control factor					
			Replicates	Extraction control	LOQ	LOD	Sample efficiency	gDNA
quantGenius	Std.curve	+	+	+	+	+	+	-
GenEx	ΔΔCq-E/Std.curve	+	+	-	-	+	-	+
qBase+	ΔΔCq-E	+	+	+	-	-	-	-
REST	ΔΔCq-E	+	+	-	-	-	-	-
DAG Expression	Std.curve	+	+	-	-	-	-	-

Quantification method, use of multiple reference genes for normalization and implementation of QC factors in quantification are compared. Std.curve – standard curve, ΔΔCq-E – efficiency corrected ΔΔCq method, replicates – replicate variability, extraction control – extraction efficiency, LOQ/LOD imputation – identification and imputation of copy numbers that are under LOQ or LOD, respectively, sample efficiency – individual sample efficiency estimate, gDNA – gDNA contamination correction

### Performance validation

The current version of the application was tested in-house for a year to detect and remove coding bugs. Further, we have analysed 50 experiments from different projects, where 40 were set on 384-well plates and 10 on the Fluidigm 48.48 Dynamic Arrays. Quantification and QC were performed in parallel in quantGenius and Microsoft Excel using preformatted formulas. A subset of the comparison is shown in the Additional files 4 and 5, respectively. Using both approaches, all the intermediate and final copy numbers, as well as those of the calculated QC parameters, were identical.

### Use cases showing the importance of the quantGenius decision support system

To show the importance of proper QC in quantitative analyses we have reanalysed two datasets from different qPCR applications using quantGenius, a gene expression study and a genetically modified organism (GMO) quantification analysis.

For the gene expression use case, a subset of qPCR data from our previously published experiment [16], analysing two target genes in the response of potato to virus inoculation. The raw data (C_q_ values) and basic experimental details are available in Additional file 6, while the experimental details are available in the original publication [16]. Three quantification approaches were compared:

- quantGenius two-dilution quantification with the default QC settings
- standard curve quantification approach without any QC-DSS
- commonly used ΔΔC_q_ approach [17], using only one dilution of the samples

**Fig. 5:**
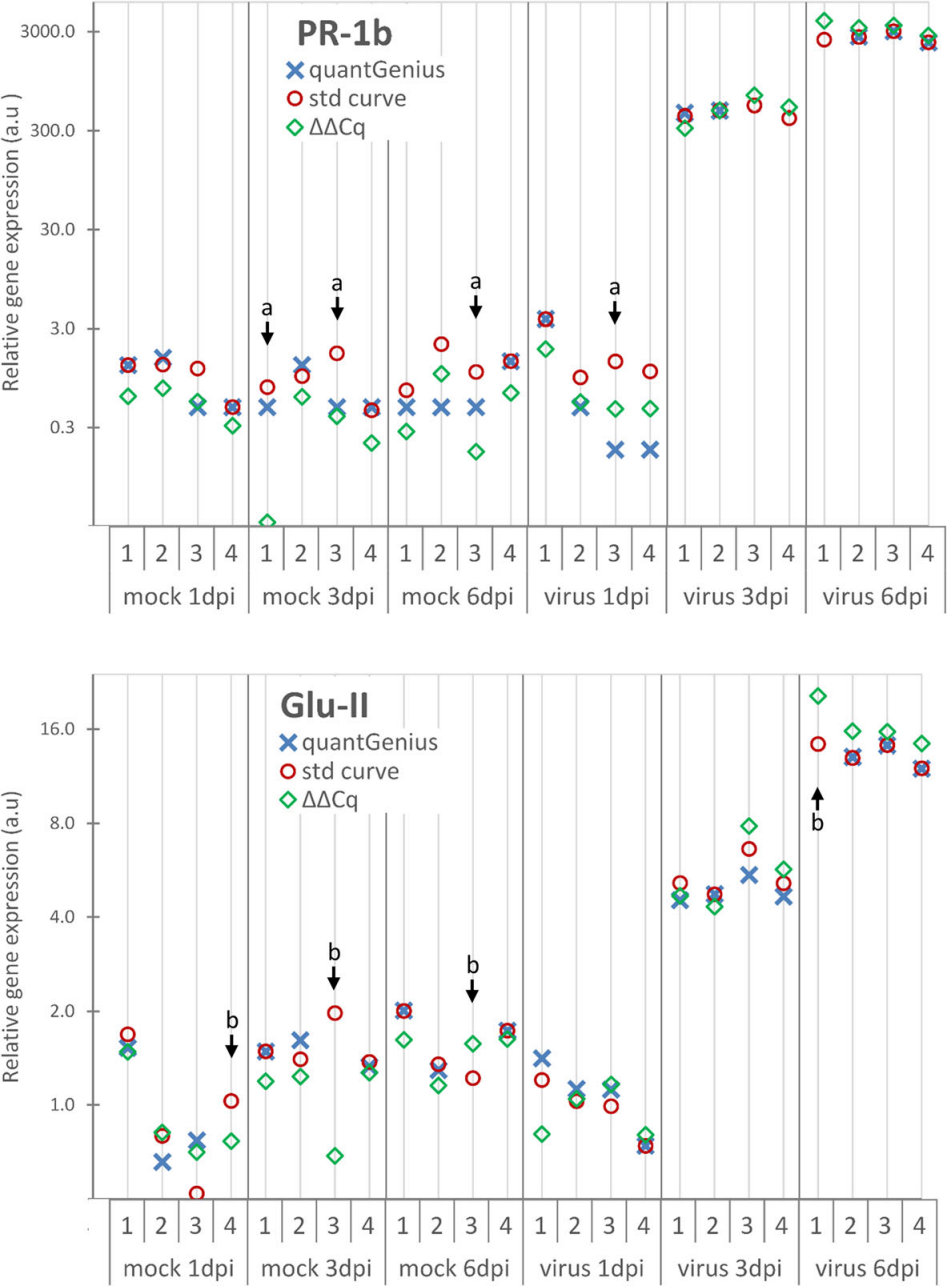
Importance of implemented QC-DSS as shown in the gene expression use case. Expression of two target genes (*PR1*-*b*, *upper panel* and *Glu*-*II*, *lower panel*) was analysed in mock- and virus-inoculated potato plants at one, three and six days post infection (dpi). *EF*-1 and *COX* were used for normalization [16], (Additional file 6). Relative expression values obtained by quantGenius (*cross*) are compared to the ones obtained using standard curve quantification without QC performed (std curve, *circle*) and the ΔΔC_q_ quantification approach (ΔΔCq, *diamond*). To get comparable values in the three approaches, the results of each approach were normalized to one of the samples (virus 3dpi 2) and then scaled to the average expression of the first experimental group (mock 1dpi). *a arrows* - examples of samples with C_q_ values near LOQ showing high variability among the quantification approaches used. *b arrows* - examples of outlier samples with an efficiency problem detected in either the target or the reference gene where results are not calculated in quantGenius

The relative copy numbers obtained in the three approaches are presented in Fig. 5 and Additional file 7. The overall results of the methods correlate highly (*r* > 0.99) for both target genes. Nevertheless, the power of quantGenius is shown in the case of individual samples with low gene expression values and sub-optimal amplification efficiencies.

The expression of the PR-1b gene was near the LOQ in the mock-inoculated samples (demonstrated as C_q_ values near C_qLOQ_ and high inter-replicate CVs), which resulted in high copy number variation (CV > 50) between different quantification approaches (Fig. 5, top panel, a arrows, Additional file 7). With quantGenius, the copy number values below LOQ are imputed with a small value number that is in the range of values calculated for other samples near LOQ. The user is alerted with a warning and will take this into account when interpreting the results. On the other hand, in the samples where only more diluted reactions were under the LOQ, only the less diluted reactions were used for the quantification.

In both target genes, there were cases of inhibition of amplification in individual samples, resulting in outlier results, which are especially evident in the Glu-II gene results (Fig. 5, b arrows). In these cases, quantGenius does not calculate the final result and thus again increases the reliability of the outputs of the quantification.

The second dataset is from the GMO diagnostics, where the quantity the GMO (Round-up-ready soybean, RRS) in the samples is quantified as a ratio of the transgene and reference gene (soybean lectin) copy numbers, both calculated from the standard curve of the reference material with known GMO content [3]. In the presented example, strong inhibition for the both, the reference gene and the transgene assays in both of the analysed DNA extractions from the same sample was observed which resulted in more diluted DNA reactions having lower C_q_ values than less diluted ones (Additional file 8). Without the QC, the calculated % of the GMO would have been ranging from 56 to 1090%, depending on the DNA isolation and dilution used. On the other hand, in the quantGenius workflow, the results for this sample are not given, primarily because of unacceptable efficiency of the reference gene (see decision tree in Fig. 4). For this sample, the DNA isolation and qPCR analysis were repeated and it then passed QC and the GMO content was determined to be 33% (data not shown).

## Discussion

The paper presents a web application for quantification of nucleic acids, integrating unique QC-based DSS (Fig. 4), built based on the acknowledged qPCR standards [1, 3, 18] which ensure that only high-quality data is used for biological interpretation. Most qPCR data analysis tools (partly reviewed in [13]) have been designed with a simple experimental design. Individual QC steps that are implemented in quantGenius are also included in other software tools (Table 1). None, however, to our knowledge, uses individual sample efficiency estimates as a QC step. Moreover, the application was built to be simple and intuitive and offers full flexibility for different experimental setups. Although the same calculations, including QC, can be done in spreadsheet software such as Microsoft Excel, the use of quantGenius does not require manual interventions for either QC or data preparation for other analysis tools. Combined with the import data consistency check-up, the use of quantGenius greatly reduces the risk of human errors when handling the data. The QC steps implemented in the DSS are the ones critical for quantification, whereas the users need to perform initial (qualitative) QC steps, such as checking fluorescence curves, qDNA contamination, the efficiency of reverse transcription, non-template or other controls, prior to importing the data to quantGenius.

quantGenius is based on quantification using a standard curve [2]. Although this approach is more robust and gives the user the biggest flexibility in the cases of suboptimal samples and/or assays [3, 6, 18] and also eliminates the need for additional interplate calibration if the same standard curve is used on all plates [3], it is implemented only in some qPCR data analysis tools (Table 1, [13]). It was previously shown that with ideal samples and assays, the results of more commonly used ΔΔCt and the standard curve approach are identical [15], as was also confirmed by the presented case study, where the correlation of the quantGenius and ΔΔC_q_ results was really high (Fig. 5, Additional file 7).

In quantGenius, normalization to several validated reference genes is enabled, as it is considered the gold standard for most of the experimental setups and is also recommended by the MIQE guidelines [1, 19]. Still, the selection and validation of the reference genes should be performed beforehand by specialized tools (GeNorm, NormFinder, BestKeeper; reviewed in [12], GrayNorm [20]).

Lower copy numbers of the reference gene can indicate problems with DNA/RNA isolation or reverse transcription yields [21], leading to unreliable quantification of target genes. The extraction control implemented in quantGenius eliminates such samples from further analyses (Fig. 4).

Depending on the biological system studied, the targets in individual samples may not be detected (are under the LOD). Moreover, low amounts of DNA in the qPCR reaction can increase the measurement uncertainty due to the high variability of quantity estimations caused by the occurrence of stochastic effects, therefore only the reactions above the LOQ can be accurately quantified [5]. The reactions where Cq values are not determined are treated differently in different analysis approaches: they are either excluded from downstream analysis, which makes further calculation impossible and can lead to unnecessary information loss or even false interpretation. Alternatively, these reactions are assigned a maximum obtained C_q_ value which leads to biased inference or they are imputed using different statistical models [22]. In the quantGenius data analysis scheme (Fig. 4), the values below LOD and LOQ are imputed taking into account the target gene copy numbers at the LOQ and average reference gene copy numbers, resembling the background correction implemented in high-throughput gene expression analysis methods [23]. Therefore, the imputed values are comparable but appropriately lower than the ones within quantification range of the assay where the LOD imputed values are lower than the LOQ imputed ones. In this way, the user can easily spot the imputed values when inspecting the resulting output matrix and take appropriate caution when interpreting such results as was shown in our gene expression use case (Fig. 5, Additional file 7). Nevertheless, in cases, where the target DNA is truly absent (e.g absence of microorganism or transgene), the LOD imputation may result in false “positive” result and in these cases the exported data matrix without the imputed values should be used for interpretation of the results.

Low reproducibility of the C_q_ values from technical replicates can be an indication of an unstable assay, a pipetting error or stochastic effects due to the low amount of DNA in the reaction [24]. The latter is implemented in quantGenius, as an indication of below LOQ target DNA amounts [6] which allows for robust analysis.

There is currently no consensus on how sample specific PCR efficiencies should be calculated and used for robust quantification. Although the individual sample amplification efficiencies determined from the amplification curves increase the random error of qPCR quantifications [24], the individual sample efficiency determination has a great value for outlier detection [25]. However, as the reaction efficiency is both sample and assay dependent [6], use of RNA spike-ins is not the best option for individual sample efficiency. Therefore, quantGenius workflow includes a simple control of PCR efficiency in individual samples by comparing the C_q_ values of two dilutions of the same sample to identify outlier samples with suboptimal efficiencies. As quantification is in those cases not accurate, no result is given for those samples. The presented approach is associated with slightly higher cost of wet-lab analysis (caa 15% higher cost for chemicals), but on the other hand it greatly increases the quality and reliability of the data, especially in samples where the presence of inhibitors is expected, such as plant samples, food and feed samples, environmental samples, microorganisms grown in complex media etc. [6, 11]. This kind of outlier samples were also observed in our gene expression dataset (Fig. 5, Additional file 7) and in the GMO quantification use case (Additional file 8). The default limits of acceptable individual sample efficiencies are quite loose, allowing for reliable detection of two-fold copy number differences. The stringency of this QC parameter can be modified depending on the application which will result in change of the quantification measurement uncertainty [3, 4]. However, in matrices free of inhibitors (e.g., cell cultures extracts, plasmid DNA), a simple (one-dilution) approach, which is also available in quantGenius, can be used safely.

To promote quantGenius use within the scientific community, the application was is registered in the ELIXIR Tools and Data Services Registry (https://bio.tools) [26]. Future improvements are envisaged to automate data import, which is especially beneficial for the analysis of data generated by high-throughput platforms. Moreover, the connection of the application database to other databases (such as gene, assay or experimental data) will contribute to data management following the FAIR Data Principles [27].

## Conclusions

As opposed to black box solutions, quantGenius was designed by biologists with ease of use, flexibility and transparency in mind. It is an intuitive and easy to use tool for qPCR data organization, analysis and decision support in various qPCR applications. The integration of QC-based DSS makes it unique and enables researchers to spend more time for interpreting the biology behind the results than analysing the data.

## Additional files

Additional file 1: Schema (ER diagram) of the database and data organization in quantGenius. (PDF 166 kb)

Additional file 2: Equations used in quantGenius workflow. (PDF 122 kb)

Additional file 3: qPCR data import screenshot. (PDF 53 kb)

Additional file 4: Example of the export of results and intermediate values for an individual gene. (XLS 41 kb)

Additional file 5: quantGenius performance validation: calculations in Excel. (XLS 50 kb)

Additional file 6 Experimental data of the gene expression use case. (XLS 71 kb)

Additional file 7: Comparison of gene expression values calculated by quantGenius and other standard methods. (XLS 49 kb)

Additional file 8: GMO quantification use case. (PDF 228 kb)

## Abbreviations

Cq: Quantification cycle
C_qExtC_: Cq value of the extraction control
C_qLOQ_: Cq value of the limit of quantification
CV: Coefficient of variation
DSS: Decision support system
GMO: Genetically modified organism
LOD: Limit of quantification
LOQ: Limit of detection
QC: Quality control
qPCR: Quantitative PCR
RRS: Round-up-ready soybean
