# Supplementary material for: quantGenius: implementation of a decision support system for qPCR-based gene quantification
Source: BMC Bioinformatics. 2017 May 25;18:276. doi: 10.1186/s12859-017-1688-7 (PMC5445471; doi:10.1186/s12859-017-1688-7)
Supplement: Supplementary file 1 — Schema (ER diagram) of the database and data organization in quantGenius. (PDF 166 kb) [file 12859_2017_1688_MOESM1_ESM.pdf]

A

B

C

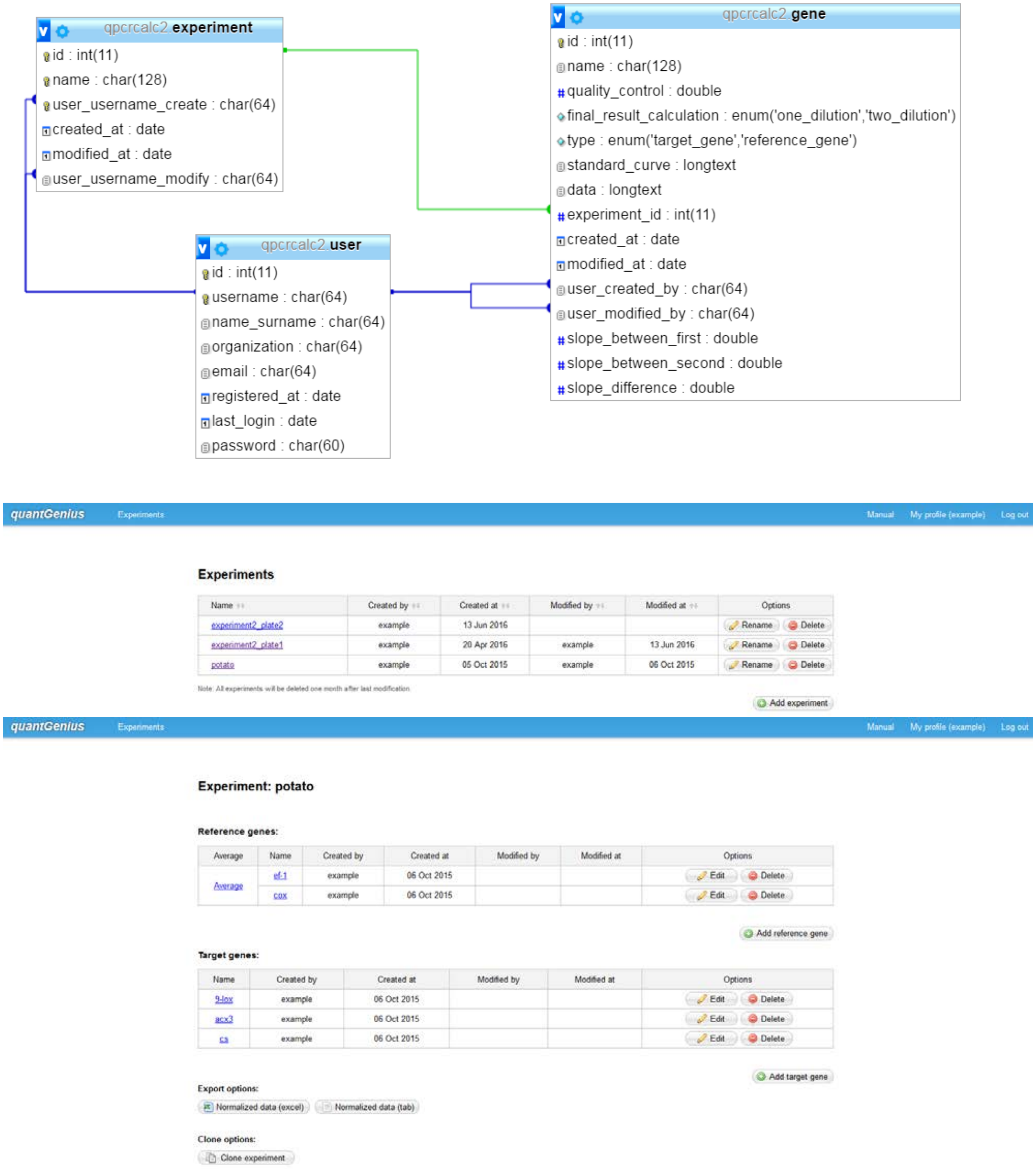

Additional file 1: Schema (ER diagram) of the database and data organization in quantGenius. A) The database is composed of 3 separate tables: “user”, “experiment” and “gene”. User data is created at registration and is connected to both, “experiment” and “gene” tables trough username. Moreover, the experiment and gene tables are connected through the experiment id. B) Experiments page: the experiments can be added, deleted renamed and sorted by name, date of creation or modification. C) Individual experiment page, the individual genes can be added, edited, or deleted. The experiment can be cloned to analyse the data with different parameter settings and experimental results can be exported in Excel (.xls) and tab-delimited (.txt) formats.
