# Supplementary material for: quantGenius: implementation of a decision support system for qPCR-based gene quantification
Source: BMC Bioinformatics. 2017 May 25;18:276. doi: 10.1186/s12859-017-1688-7 (PMC5445471; doi:10.1186/s12859-017-1688-7)
Supplement: Supplementary file 2 — Equations used in quantGenius workflow. (PDF 122 kb) [file 12859_2017_1688_MOESM2_ESM.pdf]

Additional file 2: Equations used in quantGenius workflow. For all the calculation steps, the names of the parameters (in bold), their descriptions and equations are shown. All averages are calculated as arithmetic means.

Step	Equation number, parameter name, description and equation
**Replicates**	**1** **Average C_q value**: average of the replicates (nr = number of replicates) $$\overline{Cq} = \frac{Cq_1 + Cq_2 + \cdots Cq_{nr}}{nr}$$ **2** **Replicates ΔC_q:** difference between the technical replicates (nr = number of replicates) $$\Delta Cq = \max(Cq_1, Cq_2, \ldots Cq_{nr}) - \min(Cq_1, Cq_2, \ldots Cq_{nr})$$
**Standard curve parameters**	**3** **$\log_{10}$(rel. quantity)**: logarithm with a base of 10 of the relative (arbitrary) copy number (specified in the input according to the dilution) $$log_{relQuant} = \log_{10}(\text{relQuant})$$ **4** **Slope**: regression slope of the standard curve (x = $\log_{relQuant}$; y= C_q; n = number of dilutions in the standard curve) $$slope_{std\ curve} = \frac{\sum_{i=1}^{n}(log_{relQuant\ i} - \overline{log_{relQuant}}) \cdot \sum_{i=1}^{n}(Cq_{i} - \overline{Cq})}{\sum_{i=1}^{n}(log_{relQuant\ i} - \overline{log_{relQuant}})^2}$$ **5** **Partial slope**: slope between two consecutive dilutions of the standard curve (see above) **6** **Inverse slope**: slope of the standard curve, used for copy number calculation (x = $\log_{relQuant}$; y= C_q; n = number of dilutions in the standard curve) $$inverse\ slope_{std\ curve} = \frac{\sum_{i=1}^{n}(Cq_{i} - \overline{Cq}) \cdot \sum_{i=1}^{n}(log_{rel\ quant\ i} - \overline{log_{relQuant}})}{\sum_{i=1}^{n}(Cq_{i} - \overline{Cq})^2}$$ **7** **Intercept**: intercept of the y-axis regression line $$intercept_{std\ curve} = \overline{log_{relQuant}} - inverse\ slope_{std\ curve} \cdot \overline{Cq}$$ **8** **Correlation coefficient** (rsq): square of the Pearson correlation coefficient between log concentrations and C_q values $$r^2 = \left(\frac{\sum(log_{rel\ quant} - \overline{log_{relQuant}})\ \ (Cq - \overline{Cq})}{\sqrt{\sum(log_{rel\ quant} - \overline{log_{relQuant}})^2 \sum(Cq - \overline{Cq})^2}} \right)^2$$
**Sample efficiency estimation**	**9** **Slope**: regression slope of the two sample dilutions, (x = $\log_{relQuant}$; y= C_q; dil1 = 1st dilution, dil2 = 2nd dilution) $$slope_{sample} = \frac{Cq_{dil2} - Cq_{dil1}}{log_{relQuant\ dil2} - log_{relQuant\ dil2}}$$ **10** **Slope difference**: deviation of the sample slope (Equation 9) from the standard curve slope (Equation 4) $$slope\ difference = \left| slope_{sample} - slope_{std\ curve} \right|$$
**Relative copy number calculation (each dilution separately)**	**11** **Calculated copy no.**: relative copy number of the sample based on the standard curve $$copyNo_i = 10^{(Cq_i \cdot inverse\ slope_{std\ curve} + intercept_{std\ curve})}$$ **12** **Average copy no.**: average copy number of technical replicates of the same dilution (nr = number of replicates) $$\overline{copyNo} = \frac{copyNo_1 + \cdots + copyNo_{nr}}{nr}$$ **13** **CV copy no.**: coefficient of variation of copy numbers calculated from technical replicates of the same dilution (SD – standard deviation) $$CV_{replicate\ copyNo} = \frac{SD_{copyNo_1 + \cdots + copyNo_{nr}}}{\overline{copyNo}} \cdot 100$$

Step	Equation number, parameter name, description and equation
**Dilution correction**	**14** **Dilution corrected copy no.**: takes the dilution (defined by the Rel. quantity in the data input) into account<br>$copyNo_{dilCorr} = \frac{\overline{copyNo}}{relQuant} \cdot 1000$
	**15** **CV dilutions**: Coefficients of variation of copy numbers calculated from different dilutions<br>$CV_{dil\ copyNo} = \frac{SD_{copyNo_{dilCorr}}}{\overline{copyNo_{dilCorr}}} . 100$
	**16** **Quality controlled copy no**.: calculated only for the reference genes if the QC criteria (Figure 4, step 1) are met<br>$copyNo_{QC} = copyNo_{dilCorr}$
**Normalization**	**17** **Average reference gene**: average copy number of all the samples in the experiment, calculated for each reference gene separately (k = number of samples in the experiment)<br>$\overline{reference\ gene_{\iota}} = \frac{\sum_{i=1}^{k} copyNo_{QC}}{count_{copyNo_{QC}}}$
	**18** **Scaled reference copy no.**: for each sample and reference gene, consecutive reference genes are scaled to the average of the first imported one<br>$scaled\ copy\ No_m = \frac{copyNo_{QC_m} \cdot \overline{reference\ gene_1}}{\overline{reference\ gene_{\iota}}}$
	**19** **Reference copy no.**: reference copy number used for normalization (average of scaled reference gene copy numbers for each sample)<br>$reference\ copyNo = \frac{copyNo_{QC_1} + scaled\ copyNo_2 + \cdots + scaled\ copyNo_m}{n_{ref\ genes}}$
	**20** **Normalized target copy number**: target copy number (Equation 14) normalized to reference copy number (Equation 19)<br>$normalized\ target\ copyNo = \frac{copyNo_{dilCorr}}{reference\ copyNo}$
**Final result calculation (target genes)**	**21** **Final result:** average of normalized target copy numbers (Equation 20) of both dilutions, calculated if all the condition are met (see Figure 4,steps 2-4)<br>$Final\ result = \frac{normalzed\ target\ copyNo_{dil1} + \text{normalized target}\ copyNo_{dil2}}{2}$
	**22** **CV of the normalized target copy number:** Coefficient of variation between the normalized copy numbers (Equation 20) calculated from both dilutions (SD = standard deviation)<br>$CV_{normalized\ traget\ copyNo} = \frac{SD_{normalized\ target\ copyNo}}{\overline{normalized\ target\ copyNo}} \cdot 100$
	**23** **Copy number at LOQ:** Copy number at the C_q value of the LOQ value, used for imputation<br>$copyNo_{LOQ} = \frac{10^{(\mathrm{Cq}_{LOQ} * inverse\ slope_{std\ curve} + intercept_{std\ curve})}}{relQuant_{1^{st} dilution}} \cdot 1000$
	**24** **Imputed copy number for the reactions below LOQ**<br>$Imputed\ copyNo_{LOQ} = \frac{copyNo_{LOQ}}{\overline{reference\ gene_{\iota}}} / 2$
	**25** **Imputed copy number for the reactions below LOD** (not detected)<br>$Imputed\ copyNo_{LOD} = \frac{copyNo_{LOQ}}{\overline{reference\ gene_{\iota}}} / 10$
