# Supplementary material for: quantGenius: implementation of a decision support system for qPCR-based gene quantification
Source: BMC Bioinformatics. 2017 May 25;18:276. doi: 10.1186/s12859-017-1688-7 (PMC5445471; doi:10.1186/s12859-017-1688-7)
Supplement: Supplementary file 3 — qPCR data import screenshot. (PDF 53 kb) [file 12859_2017_1688_MOESM3_ESM.pdf]

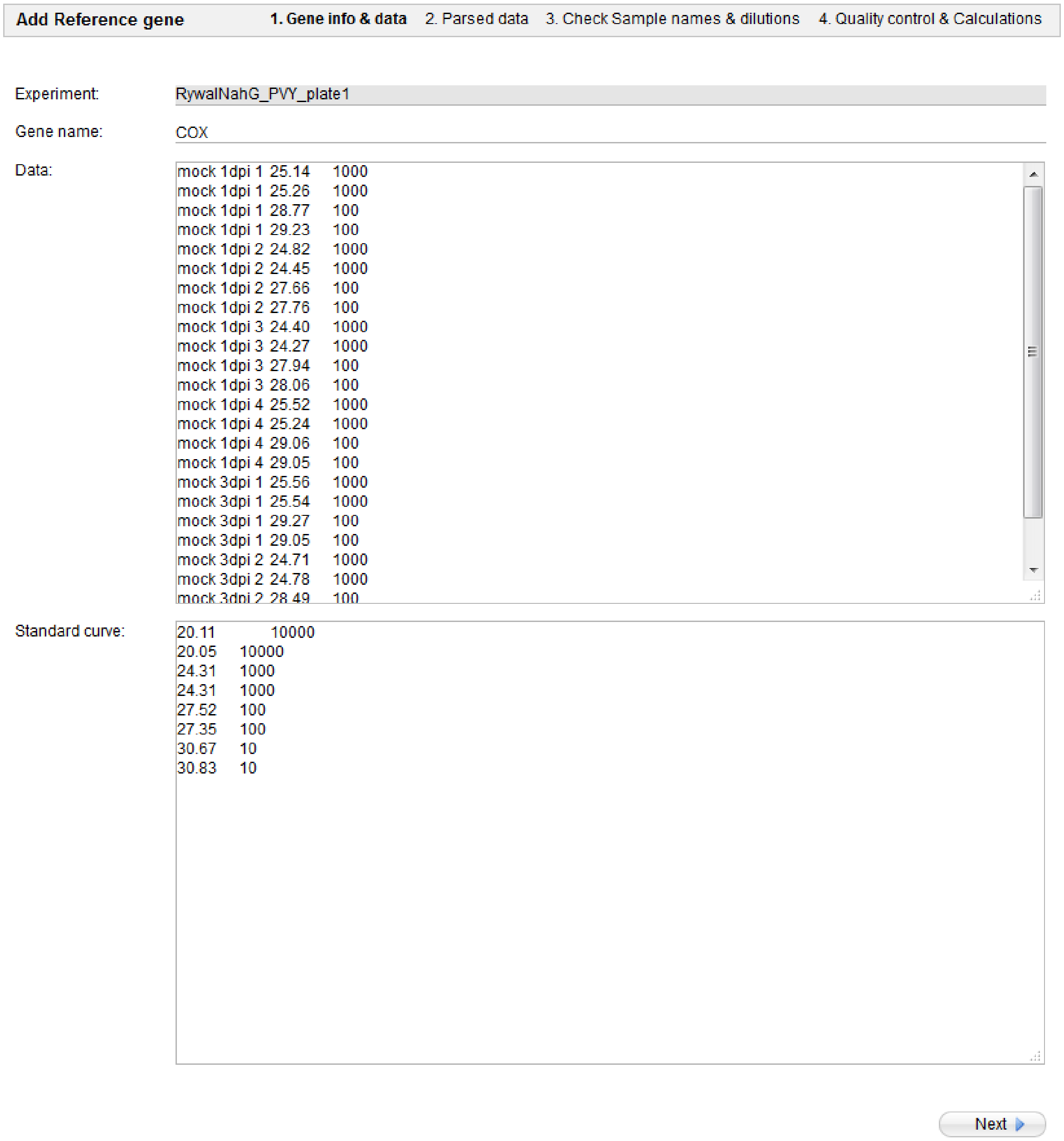

Additional file 3: qPCR data import screenshot. Data for the samples are imported in the following format: sample name [tab] C_q [tab] relative quantity. Standard curve data are imported in the following format: C_q [tab] quantity (relative for the relative quantification and absolute in the case of absolute quantification). Each line represents one qPCR reaction. In the example, each sample has two dilutions, with two technical replicates each, while there are four serial dilutions with two replicates for the standard curve.
