# Supplementary material for: quantGenius: implementation of a decision support system for qPCR-based gene quantification
Source: BMC Bioinformatics. 2017 May 25;18:276. doi: 10.1186/s12859-017-1688-7 (PMC5445471; doi:10.1186/s12859-017-1688-7)
Supplement: Supplementary file 8 — GMO quantification use case. (PDF 228 kb) [file 12859_2017_1688_MOESM8_ESM.pdf]

No quality control

Reference gene (lectin)

Involved	Sample name	Cq value	Average Cq value	Replicates Δ Cq	Rel. quantity	log (Rel. quantity)	Slope	Slope difference	Calculated copy no.	CV copy no.	Average copy no.	dilution corrected copy no.	CV dilutions	Quality controled copy no.
✓	G007/05-1	32.498783	32.4501	0.1	3	0.4771	10.8586	14.24	0.04	4.66	0.04	13.42	138.63	13.42
✓	G007/05-1	32.40136			3	0.4771			0.04					
✓	G007/05-1	27.189297	27.2692	0.16	1	0			1.42	7.64	1.34	1344.54		1344.54
✓	G007/05-1	27.349133			1	0			1.27					
✓	G007/05-2	35.091858	34.3439	1.5	3	0.4771	13.9803	17.36	0.01	66.06	0.01	4.21	140.26	4.21
✓	G007/05-2	33.59591			3	0.4771			0.02					
✓	G007/05-2	27.750986	27.6736	0.15	1	0			0.97	7.4	1.02	1022.46		1022.46
✓	G007/05-2	27.596184			1	0			1.08					

Transgene(RRS)

Involved	Sample name	Cq value	Average Cq value	Replicates Δ Cq	Rel. quantity	log (Rel. quantity)	Slope	Slope difference	Calculated copy no.	CV copy no.	Average copy no.	dilution corrected copy no.	CV dilutions	Reference copy no.	Normalized target copy no.	Final result	CV Normalized target copy no.	Warning
✓	G007/05-1	31.173443	31.2018	0.06	3	0.4771	2.7508	6.25	0.33	2.62	0.32	107.05	106.36	13.42	7.98	4.269	122.78	
✓	G007/05-1	31.230198			3	0.4771			0.32									
✓	G007/05-1	29.857807	29.8893	0.06	1	0			0.77	2.91	0.76	756.55		1344.54	0.56			
✓	G007/05-1	29.920862			1	0			0.74									
✓	G007/05-2	32.371902	32.5054	0.27	3	0.4771	4.7414	8.25	0.15	12.3	0.14	45.87	121.37	4.21	10.9	5.742	126.94	
✓	G007/05-2	32.638992			3	0.4771			0.13									
✓	G007/05-2	30.323923	30.2432	0.16	1	0			0.57	7.44	0.6	601.2		1022.46	0.59			
✓	G007/05-2	30.162497			1	0			0.63									

quantGenius (dafault QC parameters)

Reference gene (lectin)

Involved	Sample name	Cq value	Average Cq value	Replicates Δ Cq	Rel. quantity	log (Rel. quantity)	Slope	Slope difference	Calculated copy no.	CV copy no.	Average copy no.	dilution corrected copy no.	CV dilutions	Quality controled copy no.
✓	G007/05-1	32.498783	32.4501	0.1	3	0.4771	10.8586	14.24	0.04	4.66	0.04	13.42	138.63	Explain
✓	G007/05-1	32.40136			3	0.4771			0.04					
✓	G007/05-1	27.189297	27.2692	0.16	1	0			1.42	7.64	1.34	1344.54		Explain
✓	G007/05-1	27.349133			1	0			1.27					
✓	G007/05-2	35.091858	34.3439	1.5	3	0.4771	13.9803	17.36	0.01	66.06	0.01	4.21	140.26	Explain
✓	G007/05-2	33.59591			3	0.4771			0.02					
✓	G007/05-2	27.750986	27.6736	0.15	1	0			0.97	7.4	1.02	1022.46		Explain
✓	G007/05-2	27.596184			1	0			1.08					

Transgene(RRS)

Involved	Sample name	Cq value	Average Cq value	Replicates Δ Cq	Rel. quantity	log (Rel. quantity)	Slope	Slope difference	Calculated copy no.	CV copy no.	Average copy no.	dilution corrected copy no.	CV dilutions	Reference copy no.	Normalized target copy no.	Final result	CV Normalized target copy no.	Warning
✓	G007/05-1	31.173443	31.2018	0.06	3	0.4771	2.7508	6.25	0.33	2.62	0.32	107.05	106.36	/	/	/	/	QC ref failed
✓	G007/05-1	31.230198			3	0.4771			0.32									
✓	G007/05-1	29.857807	29.8893	0.06	1	0			0.77	2.91	0.76	756.55		/	/			
✓	G007/05-1	29.920862			1	0			0.74									
✓	G007/05-2	32.371902	32.5054	0.27	3	0.4771	4.7414	8.25	0.15	12.3	0.14	45.87	121.37	/	/	/	/	QC ref failed
✓	G007/05-2	32.638992			3	0.4771			0.13									
✓	G007/05-2	30.323923	30.2432	0.16	1	0			0.57	7.44	0.6	601.2		/	/			
✓	G007/05-2	30.162497			1	0			0.63									

Additional file 8: GMO quantification use case. Two DNA extractions of the unknown sample (G007/5-1 and G007/5-2) were analyzed for the quantity of the transgene (RRS) in comparison to the reference gene (lectin) copy numbers. The reactions were set as described by Cankar et al. (2006) with two dilutions, each with two technical replicates for each of the isolations. The standard curve of the reference material with 5% RSS was set with 6 serial dilutions for the lectin and 4 for the RSS, each with three technical replicates. The data was analyzed without any QC (top panel) and using quantGenius default QC parameters (bottom panel). Because of vast inhibition, the results calculated from different dilutions differ tremendously (red arrow). Using quantGenius DSS, the efficiency problems are exposed by highlighting the QC parameters that are out of predefined thresholds (orange arrows). The reference gene copy numbers and final results are not given and a warning is issued (blue arrows).
